# Prevalence of Depression and Fish Consumption among First Year Spanish University Students: UniHcos Project

**DOI:** 10.3390/nu15122757

**Published:** 2023-06-15

**Authors:** María Morales-Suárez-Varela, Carmen Amezcua-Prieto, Agustín Llopis-Gonzalez, Carlos Ayan Perez, Ramona Mateos-Campos, Natalia Hernández-Segura, Rocío Ortiz-Moncada, Ana Almaraz, Juan Alguacil, Miguel Delgado Rodríguez, Gemma Blázquez Abellán, Jéssica Alonso-Molero, Virginia Martínez-Ruiz, Irene Santana-Garcia, José M. Cancela, Luis Félix Valero Juan, Sandra Martín-Peláez, Tania Fernández-Villa, Grupo de Investigación UniHcos

**Affiliations:** 1 Research Group in Social and Nutritional Epidemiology, Pharmacoepidemiology and Public Health, Department of Preventive Medicine and Public Health, Food Sciences, Toxicology and Forensic Medicine, Faculty of Pharmacy, Universitat de València, 46100 Burjassot, Spain; agustin.llopis@uv.es (A.L.-G.); isangar@alumni.uv.es (I.S.-G.); 2 CIBER of Epidemiology and Public Health, Carlos III Health Institute, 28029 Madrid, Spain; carmezcua@ugr.es (C.A.-P.); alguacil@dbasp.uhu.es (J.A.); mdelgado@ujaen.es (M.D.R.); virmruiz@ugr.es (V.M.-R.); sandramartin@ugr.es (S.M.-P.); tferv@unileon.es (T.F.-V.); 3 Department of Preventive Medicine and Public Health, Universidad de Granada, 18016 Granada, Spain; 4 Instituto de Investigación Biosanitaria ibs, 18012 Granada, Spain; 5 Well-Move Research Group, University of Vigo, 36310 Vigo, Spain; cayan@uvigo.es; 6 Area of Preventive Medicine and Public Health, Department of Biomedical and Diagnostic Sciences, Universidad de Salamanca, 37007 Salamanca, Spain; rmateos@usal.es (R.M.-C.); luva@usal.es (L.F.V.J.); 7 Area of Preventive Medicine and Public Health, Department of Biomedical Sciences, Universidad de León, 24071 León, Spain; nhers@unileon.es; 8 Food and Nutrition Research Group, Department of Community Nursing, Preventive Medicine and Public Health and History of Science, Universidad de Alicante, 03690 San Vicente del Raspeig, Spain; rocio.ortiz@ua.es; 9 Preventive Medicine and Public Health, Faculty of Medicine, University of Valladolid, 47005 Valladolid, Spain; aalmaraz@med.uva.es; 10 Natural Resources, Health and Environment Research Center (RENSMA), University of Huelva, 21071 Huelva, Spain; 11 Department of Preventive Medicine and Public Health, University of Jaen, 23071 Jaén, Spain; 12 Area of Preventive Medicine and Public Health, Department of Medical Sciences, School of Pharmacy, Universidad de Castilla-La Mancha, 02071 Albacete, Spain; gemma.blazquez@uclm.es; 13 School of Medicine, Universidad de Cantabria, 39005 Santander, Spain; alonsomoleroj@gmail.com; 14 Marqués de Valdecilla Research Institute-IDIVAL, 39011 Santander, Spain; 15 HealthyFit Research Group, Faculty of Education and Sports Sciences, Universidad de Vigo, 36005 Pontevedra, Spain; chemacc@uvigo.es; 16 Group of Investigation in Interactions Gene-Environment and Health (GIIGAS), Institute of Biomedicine (IBIOMED), Universidad de León, 24071 León, Spain

**Keywords:** fish intake, depression, university students, diet quality

## Abstract

The World Health Organization estimates that one fifth of university students have experienced major depressive disorder at some point in their lives. Nutrition may be one of the modifiable factors that influence the development of depression. Specifically, low omega-3 fatty acid and vitamin D levels, both nutrients found in high quantities in fish, have been linked to depressive disorders. The main objective of this study was to evaluate the prevalence of depression among young Spanish university students, in addition to the pattern of fish consumption among students and the possible relationship between fish consumption and the presence of depression. Data were collected retrospectively from a nationally representative sample of 11,485 Spanish university students aged 18 years or older in 11 Spanish universities, from 2012 to 2022. The respondents were analyzed according to frequency of consumption and compliance with weekly recommendations for fish intake and the presence of depression. Regression models were also performed to determine students’ odds of depression as a function of compliance with recommendations according to selected sociodemographic variables. The prevalence of depression was 10.5%; it was more prevalent in women, older students and in those with both high and low BMIs. In addition, it was also more prevalent in those that lived outside the family home, with roommates and those who were employed. Sixty-seven percent of the students met the fish intake recommendations. The most common frequency of fish consumption was 1–2 times/week (44.2%), and the least frequent was 2.3% daily fish consumption. Students from northern universities were more likely to consume fish (68.4%) than those from southern universities (66.4%). Non-consumption of fish was found to increase the risk of depression (ORa = 1.45 (1.28–1.64); AF = 31.0% (21.9–39.0)), but it was the student’s own conditions that had the greatest influence on the development of the disorder. In summary, a lower consumption of fish seems to be associated with a higher incidence of depression in Spanish university students; however, other social factors of the student may influence the development of the disorder, and all of this should be taken into account for the development of prevention strategies.

## 1. Introduction

According to the World Health Organization (WHO), depression is a mental disorder characterized by a persistent feeling of sadness, lack of pleasure or interest in general [1]. This affects the ability to carry out daily activities in the workplace, school, or family, and, therefore, depression is considered a cause of disability. It is estimated that around 3.8% of the world’s population suffers from this disorder, and this number has been increasing over the years [1]. This is particularly concerning because, in addition to being a cause of disability, in more severe cases, depression can lead to death by suicide. In fact, suicide is the most common cause of death in younger age groups [1].

This disorder does not have a single cause, but rather different circumstances in a person’s life that lead to the disorder [1]. There are risk factors that can lead a person to develop this disorder, mainly life circumstances such as grief, trauma, abuse, poor economic situation, etc., that often trigger the disorder. However, genetic and behavioral factors also play a role in its development [2]. According to some studies, the physiological mechanisms by which the disorder develops are very broad, involving the signaling systems of the central nervous system, neurotransmitters, receptors, as well as other systems such as the endocrine or even the immune system [3].

For university students, according to a study by the WHO’s World Mental Health International College Student Project, which estimated the prevalence of some mental disorders, 21% of those surveyed had experienced major depressive disorder at some point in their lives, while 18% had experienced it in the past year [4].

In addition, it is estimated that, during the COVID-19 pandemic, the prevalence of mental disorders in young people has doubled compared to previous years [5,6]. There are numerous studies that show that the COVID-19 pandemic has had a negative psychological effect on college students and the proportion showing mental health problems is alarming [7,8,9,10,11]. Governments enforced strict social distancing measures, including large-scale lockdowns and the closures of educational institutions, leading to isolation and reduced social contact. These measures led to unprecedented disruptions in people’s daily lives while also placing a significant mental health burden [11]. Negative mental health effects had also been previously seen in past containment efforts during infectious disease outbreaks [12,13,14,15,16].

University students are exposed to situations that can influence the development of mental disorders, such as academic pressure, schedules, new friendships, economic factors, change in usual residence, etc. Mental disorders during the university stage can have both short-term effects, as they influence academic performance, as well as medium- to long-term effects, as they can have repercussions on the normal development of adult life, affecting performance and personal relationships [17,18].

Nutrition can be one of the factors that influences the development of depression. On the one hand, a good diet provides the necessary nutrients for the proper functioning of the brain while, on the other hand, unhealthy dietary patterns and the intake of certain nutrients that can be harmful and can negatively impact the development of depressive disorders [19]. However, while the intake of certain nutrients, or tendencies towards certain dietary patterns, have been related to the improvement or aggravation of depression, there is no clear evidence of how diet influences it [20]. The Mediterranean diet is one of the patterns that has shown the best positive effects against depression, and greater adherence to it may be related to a lower risk of experiencing depressive symptoms [19,21]. Generally speaking, diets with a higher intake of unhealthy fats, such as trans fats, alcohol consumption, etc., can cause damage to the brain, as these nutrients can produce effects such as inflammation or oxidative stress that increase the risk of experiencing depressive symptoms [19].

Spain, due to its geographical location, has always been marked by Mediterranean culture. Within the Mediterranean diet, one of the most prominent foods is fish and, in Spain, this product is particularly important because it is one of the main producers and one of the European countries with the highest fish consumption [22]. However, in recent years there has been a significant decrease in fish consumption, at the same time as frozen and processed meat consumption has increased [23].

In the Spanish geography, the northern communities have a higher fish consumption, with Galicia, Castilla y León, Asturias, and the Basque Country, among others, having a higher per capita fish consumption. On the other hand, communities such as Extremadura, Valencia, and Andalusia are below the Spanish average [24].

Some nutrients commonly found in high quantities in fish have been linked to depressive disorders. Fish is one of the main dietary sources of omega-3 polyunsaturated fatty acids (PUFAs). Omega-3 PUFAs are synthetized by dietary shorter-chained omega-3 fatty acid alpha-linolenic acid (ALA) to form the more important long-chain omega-3 fatty acids: eicosapentaenoic acid (EPA) and docosahexaenoic acid (DHA) [25]. Omega-3 PUFAs have been considered of particular interest for the treatment of certain forms of chronic diseases [26] and many epidemiological and experimental studies have emphasized their possible role in the prevention or treatment of depressive disorders. In previous studies, these components have been found in lower levels in depressive patients [21]. Due to evidence from animal and human studies reporting that omega-3 deficiency leads to impaired neuronal function (especially of serotoninergic and dopaminergic neurotransmitters) and altered inflammatory status, the biological plausibility of the effects of the omega-3 PUFAs raised several hypotheses, although they are merely speculative [27]. Although epidemiological data and clinical trials suggest that omega-3 PUFAs may have preventive and therapeutic effects on depression, the underlying mechanisms are still unclear. The protective role of omega-3 fatty acids against depression has been hypothesized to depend on the physiological mechanisms in which fatty acids take part.

A recent umbrella review found moderate-quality evidence suggesting that a 100 g per day increment in fish consumption is associated with a lower risk of depression (SRR: 0.88; 95% CI: 0.79, 0.98) and a meta-analysis of interventional studies indicated that supplementation with EPA and DHA may be effective in reducing depressive symptoms [28]. The results of another systematic review and meta-analysis of dietary patterns and depression suggested that high intakes of fish may be associated with a reduced depression risk which indicates that dietary interventions have the potential to be included as a primary prevention strategy for depressive disorder [29]. A systematic review and meta-analysis of observational studies found that a pooled analysis of the highest vs. the lowest (reference) fish consumption category resulted in a 22% decreased risk of depression (RR: 0.78; 95% CI: 0.69, 0.89) and specific dose analyses revealed that a significant decreased risk was reached at a 50 g per day intake (RR: 0.84; 95% CI: 0.72, 0.99) [30]. There is, however, evidence to support the lack of association between fish intake and incident depression (OR: 1.00; 95% CI: 0.80, 1.26), but it must be noted that the population studied here were middle-aged and older adults [31].

Regarding specific nutrients, a systematic review and meta-analysis of observational studies supported the hypothesis that dietary omega-3 PUFA intake is associated with a lower risk of depression [30]. The analysis of the extreme categories of omega-3 PUFA intake and depression resulted in a significantly reduced risk of depression (RR: 0.82; 95% CI: 0.73, 0.92) and the dose–response analysis demonstrated a decreased risk of depression up to 1.8g per day of omega-3 PUFA intake (RR: 0.30; 95% CI: 0.09, 0.98) [30].

Another nutrient potentially related to depression and/or mental health problems present in fish is vitamin D. Vitamin D is a fat-soluble vitamin, whose main precursors are Vitamin D3 (cholecalciferol) and D2 (ergocalciferol) [32]. Vitamin D3 can either be obtained from the diet, or it can be synthesized upon sun exposure of the skin [32]. Recently, researchers found that there is a slight link between vitamin D and depression; however, the link is not completely understood [33]. Additionally, this link does not prove whether a low level of vitamin D causes depression or if depression causes a low level of vitamin D. The only certainty is that the risk of depression may be further exacerbated by low serum levels of vitamin D. An accumulating number of studies have indicated that vitamin D also acts as a neuroactive steroid, [34,35] which plays a key role in the expression of neurotransmitters, the regulation of neurotrophic factors, neuroimmunomodulation, the production of antioxidants and neurotropic factors, making it biologically plausible that vitamin D might be associated with depression.

A systematic review aimed at examining whether vitamin D deficiency or insufficiency is associated with depression showed that empirical studies appear to provide increasing evidence for an association between vitamin D insufficiency and depression [36]. The overall findings of another review of both original studies and reviews/meta-analysis papers were that there is a relationship between vitamin D and depression, though the directionality of this association remains unclear, and the association appears to be driven by the homeostatic, trophic, and immunomodulatory effects of vitamin D [37].

While it seems clear that serum vitamin D levels inversely correlate with clinical depression, the currently available evidence is not strong enough to recommend universal supplementation in depression [37]. Evidence from supplementation trials suggest a more robust therapeutic effect on subjects with major depression and concurrent vitamin D deficiency [36,37]. A meta-analysis of randomized controlled trials without biological flaws demonstrated a statistically significant improvement in depression with Vitamin D supplements (0.78; 95% CI: 0.24, 1.27). Studies with biological flaws were mainly inconclusive, with the meta-analysis demonstrating a statistically significant worsening in depression by taking Vitamin D supplements (−1.1; 95% CI: −0.7, −1.5) [38]. Vitamin D supplementation (≥800 I.U. daily) was somewhat favorable in the management of depression in studies that demonstrate a change in vitamin levels, and the effect size was comparable to that of anti-depressant medication [38]. However, there are conflicting studies and the result of a meta-analysis of randomized controlled trials indicated that the oral administration of vitamin D did not have a significant effect on the reduction in post-intervention depression scores with a standardized mean difference for the pooled data of −0.91 (95% CI: −2.02–0.19) [39].

Previous studies have observed that the diet of young people is moving away from Mediterranean patterns and adopting characteristics of the Western diet, characterized by a high consumption of sugars and saturated fats, a pattern that has shown pro-inflammatory effects related to depressive symptoms [5].

According to a study by Ramón-Arbúes et al. [40], which analyzed the diet of university students in relation to some mental health disorders, the prevalence of inadequate nutrition among the university population was particularly alarming, with around 82% of the studied population having poor eating habits. It was identified that insufficient consumption of certain foods, such as lean meats and fish, showed a relationship with depressive symptoms [40]. In another study by Hilger et al., most of the surveyed students reported having had changes in their diet when starting university, and these changes particularly affected meat and fish consumption [41].

The main objective of this study was to determine the prevalence of depression among Spanish university students, as well as their fish consumption patterns and the possible relationship between fish consumption and the presence of depression.

## 2. Materials and Methods

This cross-sectional, nested case-control study is part of the UniHcos project [42], a multicenter study of versatile prospective cohorts involving 11 Spanish universities (León, Vigo, Jaen, Granada, Salamanca, Huelva, Alicante, Cantabria, Valladolid, Castilla-La Mancha, and Valencia) aimed at understanding students’ lifestyle habits upon entering university and how they change during their stay. The UniHcos project has been approved by the Ethics Committees of the collaborating universities, and the data file integration complies with the European and Spanish laws on the protection of personal data.

The data for this study were collected in the academic years 2012–2022. Information on fish consumption and personal characteristics was collected via an online self-administered questionnaire completed by all students who met the selection criteria and agreed to participate in the project. The online questionnaire also included informed consent and ethical approval. The main inclusion criteria were being a first-year student and first enrolment across all degrees of each participating university.

**Figure 1:**
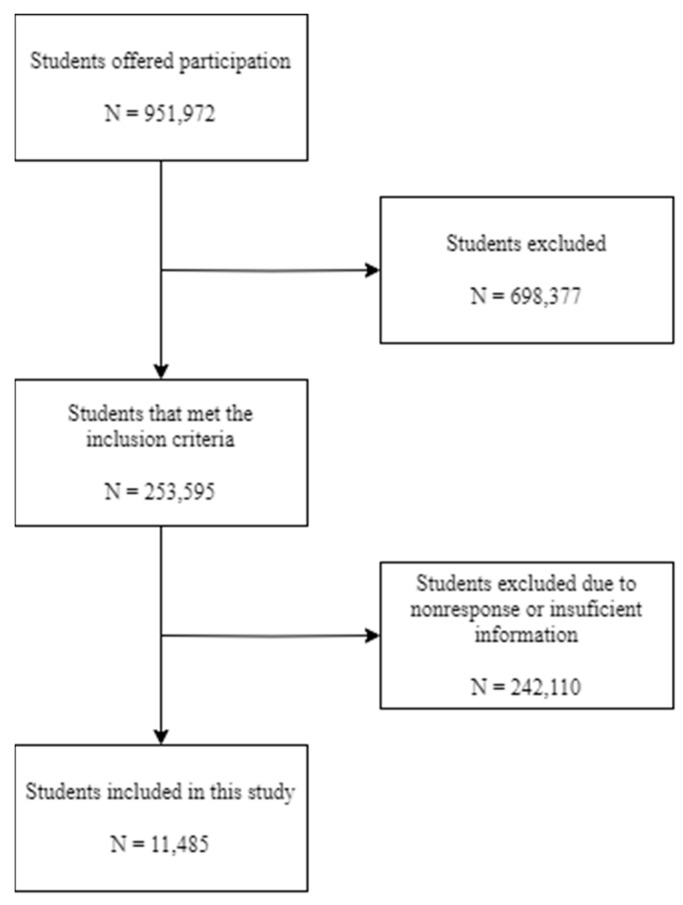
Flow diagram of the recruitment and enrollment of the studied sample.

A flowchart detailing the recruitment process is included, starting with the number of students that offered to participate and ending with the number finally included in this study (Figure 1).

According to the authors’ experience, low participation rates in similar research among first-year university students are common. This results in unit nonresponse bias, which occurs when there is a difference between the characteristics or responses of the individuals invited to participate and those of the subjects ultimately included in the study. To avoid this as much as possible, the questionnaire was designed to avoid making it more likely for certain groups to participate or not in the study. It was sent via institutional email and students were given sufficient time and reminders to respond, in addition to guaranteeing confidentiality.

It was not possible to measure and adjust for nonresponse bias through weighting adjustments, post-stratification, or propensity models due to a lack of sufficient demographic or database variables. Nonresponse bias to the item, which occurs when a respondent does not answer certain questions in a survey, was not a concern, as students who did not give sufficient information were excluded from the study.

The personal and sociodemographic variables collected were: gender (male, female); age (years); body mass index (BMI) (˂18.5: underweight, 18.5–24.9: normal weight, 25–30: overweight, >30: obesity); marital status (single, de facto partner, married, separated/divorced (calculated by summing separated and divorced individuals), widowed); employment status (not seeking employment and studying, studying and seeking employment, studying and working part-time, studying and working full-time); housing, defined as the place where the student lives during the academic year (family home, dormitory/university residence, rental, own home/other); and living arrangements, defined as the people with whom the student lived during the academic year (with parents, roommates/friends, with partner, with children, alone). For data interpretation, variables were recategorized as follows:

- Employment status: Unemployed (studying and not seeking employment, studying and seeking employment), employed (studying and working part-time, studying and working full-time);
- Living arrangements: Parents, roommates (roommates/friends), partner (with partner, with children), alone. To facilitate data comparison, those who lived with others were grouped into the “accompanied” category;
- Location: Universities in the north (Cantabria, Castilla-La Mancha, León, Vigo, Salamanca, and Valladolid) and universities in the south (Granada, Jaén, Huelva, Alicante, and Valencia);
- Marital status: The categories of “separated” and “divorced” were combined.

The dependent variable was constructed based on responses to the food frequency consumption section (FFCS) of the online self-administered questionnaire completed by all students, following the model of question 96 in section H4 of the Spanish National Health Survey of 2006, which had been previously validated. To estimate fish intake, information on consumption was collected through a question where participants could select the option that best suited them, with options of never/rarely, <1 time per week, 1–2 times per week, 3–4 times per week, or daily. No information was collected on serving size or quantity of fish consumed.

To evaluate factors associated with fish consumption, the recommendations established in the Mediterranean Diet Foundation pyramid [43] were taken as the gold standard, where the recommended consumption of fish is 2 or more servings per week. For the interpretation of the results, consumption frequencies were grouped into “compliant” or “non-compliant” with the Mediterranean Diet Foundation recommendations. Students who complied with the recommendations were classified as those who consumed fish 1–2 times/week, 3–4 times/week, or daily. On the other hand, the “non-compliant” group was composed of those who never or rarely consumed fish or those who consumed it less than once a week.

To collect information about depression, participants were asked whether they had been diagnosed with depression by a professional.

There is no information available about the time of year when the data were collected. The completed dietary questionnaire was coded and entered into the database for this study.

### Statistical Analysis

Firstly, the students were classified according to the origin of their university. The presence of depression was also analyzed according to the sociodemographic characteristics of the population. The Chi-square test was performed with a statistical significance level of *p* < 0.05 to check if there was a relationship between the groups. Next, the sample was also analyzed according to compliance with the recommended fish consumption, evaluating both the frequency of consumption and the frequencies grouped according to whether they complied with the recommendation or not, based on the variables of origin. Finally, compliance with fish consumption recommendations was studied in depressed and non-depressed individuals, based on sociodemographic characteristics. Binary logistic regression techniques (OR) and 95% confidence intervals (CI) were calculated, stratified by sociodemographic variables, to determine the factors contributing to students suffering from depression. A fish consumption ratio was also calculated to determine the adjustment to the average intake of students based on per capita fish consumption by the autonomous communities to which each university belongs. This was calculated by dividing the percentage of people who complied with the recommendations in each province by the per capita consumption of each community [24]. All analyses were performed using survey routines and dietary survey weights to maintain the nationally representative nature of the data. Data analysis was performed using IBM-SPSS statistical package version 20.0 (IBM Corp. Released 2011. IBM SPSS Statistics for Windows, Version 20.0. IBM Corp., Armonk, NY, USA).

## 3. Results

### 3.1. General Characteristics of Surveyed Students

This study involved 11 universities throughout the Spanish territory, in which the participation rate of students was uneven (Table 1). The universities in the southern regions had the highest participation rates, accounting for around 60% of the sample, with the University of Granada having the highest response rate (25.9%), followed by Valencia (17.9%). On the other hand, although more universities located in the north were included in the study, there was less participation, with only 40% of participants coming from the north. The universities with the lowest participation rates were Cantabria and Castilla-La Mancha (0.9% and 2.6%, respectively).

### 3.2. Characteristics of Students

As shown in Table 2, a total of 11,485 students participated in the study, of whom 72.7% were female. The overall mean age was 20.1 years, with the mean age of women being slightly lower than that of men, at 19.97 and 20.47 years, respectively. It is worth noting that around 52% of students were 18 years old or younger.

As for anthropometric characteristics, 71.6% of surveyed students were in the normal weight range, while almost 20% were overweight or obese and around 10% were underweight.

Just under half of the participants (45.4%) lived in their family home, but a large percentage lived in rented flats or university residences (11.3% and 39.3%, respectively). Therefore, the majority lived with their parents (42.2%) or flatmates (42.5%).

Regarding marital status, the vast majority of students were single (90.6%), followed by those who had a common-law partner (7.3%).

With regard to employment, most respondents (66.3%) were only studying, while 22.9% were combining their studies with job searching, and only 10.7% were working while studying, mainly part-time (8.1%).

Of the total sample, 10.5% of the students were diagnosed with depression by a professional, with women having a higher prevalence of the disorder compared to men.

It is also noteworthy that the mean age was higher in depressed participants (21.02 years ± 5.2) compared to non-depressed participants (20 years ± 4.5). Even when analyzing age by gender, the same trend was observed. In women, the difference remained one year (20.97 in depressed women ± 5.27 versus 19.83 ± 4.23 in non-depressed women), while in men, the difference was slightly smaller (21.28 ± 5.03 in depressed men versus 20.42 ± 5.18 in non-depressed men).

Regarding anthropometric characteristics, it was significant that depressed students had a higher prevalence of both underweight and obesity, while non-depressed students had a higher prevalence of normal weight.

Depressed students tend to live outside their family home, although this trend was not significantly different, particularly for those living in rental flats. A higher proportion of depressed students had their own house.

Regarding marital status, significant differences were only found among separated/divorced students, although married students also tended to be more depressed, albeit without significant differences. Students who only studied and were not seeking work had significantly less depression, however, students who combined studying with work were more depressed.

### 3.3. Description of Fish Intake According to the Distribution of Students

**Figure 2:**
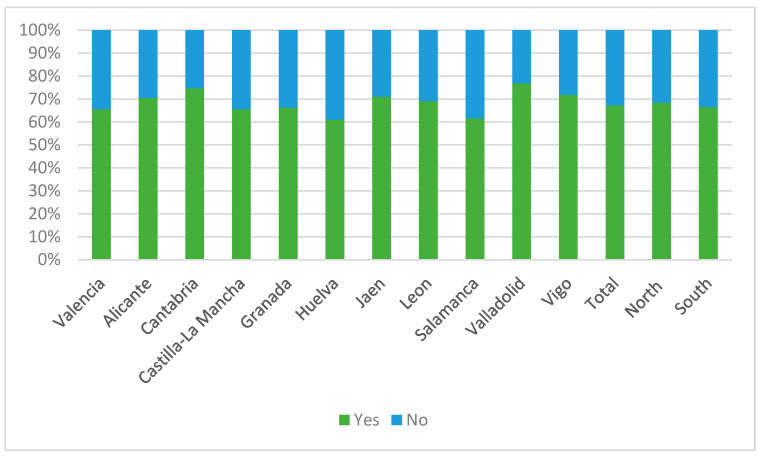
Compliance with recommendations on the frequency of consumption of fish by universities and according to geographical distribution.

**Figure 3:**
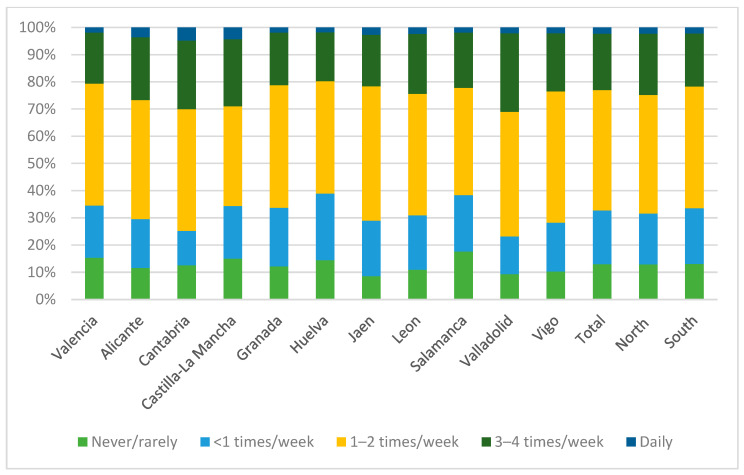
Frequency of consumption of fish by universities and according to geographical distribution.

Figure 2 and Figure 3, and Supplementary Table S1, detail fish consumption among the students according to their university of enrollment. Over two-thirds of the students (67.2%) met the recommendations for weekly fish intake. It was more common for students to consume fish 1–2 times a week compared to other frequencies, and only 2.3% of students consumed fish daily. Among the group of students who did not meet the recommendation, it was more common for them to consume fish less than once a week rather than never.

When analyzing the consumption patterns of each university individually, a similar fish consumption pattern was found in all universities, where it was significantly more frequent to meet the intake recommendation, and it was more common among those who met the recommendation to consume fish 1–2 times a week. On the other hand, in the universities of Granada, Huelva, Jaén, León, Salamanca, Valladolid and Vigo, students who did not meet the recommendation were significantly more likely to consume fish less than once a week compared to never or rarely consuming it.

Compared to other universities, Huelva and Salamanca had a lower tendency to meet the recommendation, while Cantabria and Valladolid were the universities where the most students met the recommendation. Additionally, in Cantabria and Castilla-La Mancha, there was a higher proportion of students who consumed fish daily. On the other hand, Salamanca and Alicante were the universities with the highest percentage of students who never consumed fish.

When grouped together, there was a higher proportion of students in the northern universities who met the recommendation, and it was more frequent in this region for students to consume fish 3–4 times a week or daily compared to southern students.

Furthermore, based on fish consumption in the autonomous communities of each university, it was the southern universities that were more in line with their community’s per capita consumption, with Valencia, Alicante, and Jaén being the universities that best matched the per capita consumption. However, it was in Galicia (where the University of Vigo is located) and Castilla y León (where the Universities of León, Salamanca, and Valladolid are located) where there was a higher per capita consumption. Meanwhile, in Andalusia (which includes the Universities of Granada, Huelva, and Jaén) and the Valencian Community (where the Universities of Alicante and Valencia are located), there was a lower per capita consumption.

### 3.4. Compliance with Fish Consumption Recommendations in Depressed and Non-Depressed Individuals Based on Population Characteristics

**Figure 4:**
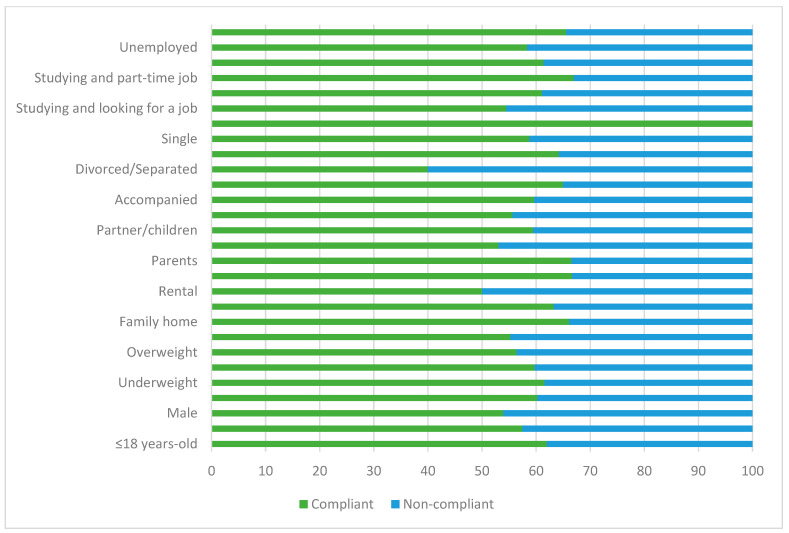
Compliance with fish consumption recommendations in depressed students based on population characteristics.

**Figure 5:**
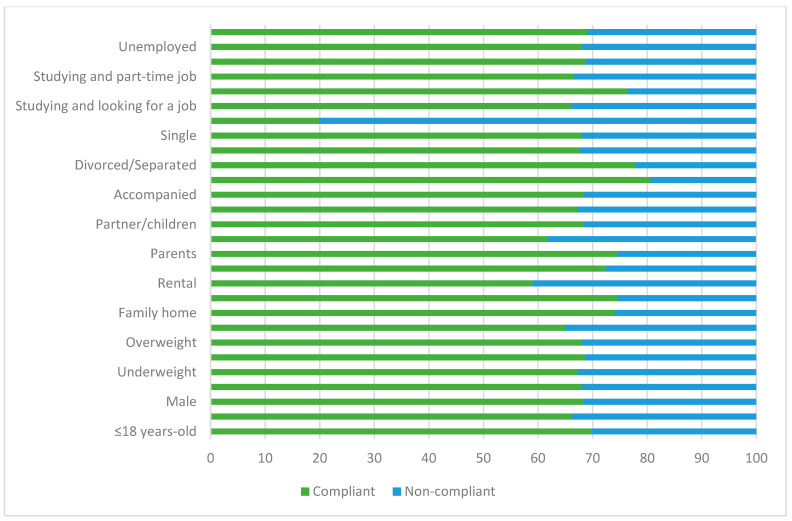
Compliance with fish consumption recommendations in non-depressed students based on population characteristics.

Figure 4 and Figure 5, and Supplementary Table S2, show the compliance rate with recommended weekly fish intake of the students according to their depression diagnosis status and personal characteristics. In general, both depressed and non-depressed students were likely to comply with fish consumption recommendations. However, there was less tendency for depressed individuals to comply with recommendations compared to non-depressed individuals.

On the other hand, there were no significant differences between depressed students who lived in rented accommodation, lived with flatmates, and those who lived alone, as they showed less difference between those who complied with recommendations and those who did not compared to the rest of students. It was also observed that the difference was smaller for those who studied and at the same time as looking for a job. Additionally, the group of employed students, especially the group of depressed students, tended to consume more fish.

### 3.5. Analysis of the Evaluation of the Influence of Fish Intake According to Population Characteristics

The risk of depression diagnosis due to inadequate weekly fish intake is shown in Table 3. In the crude model, non-compliance with fish recommendations elevated the risk of depression by 47% with an attributable fraction of 32%. In order to account for the possible influence of other factors, adjusted models were also performed and the results remained the same. The saturated model showed an increase in the risk of depression diagnosis of 45% with an attributable fraction of 31%, which was very similar to the results of the crude model. Therefore, the variables that were adjusted for in the model appear to not have a significant influence on the risk of depression diagnosis in this particular study.

## 4. Discussion

In this study, 10.5% of the surveyed students had been diagnosed with depression, which is slightly lower than the prevalence of depressive symptoms found in Arbues et al.’s study, where the prevalence was almost 20% for both men and women. This difference could be due to the fact that, while their study evaluated the presence of depression through a self-administered questionnaire, our study only took into account those who had been diagnosed by a professional, potentially leaving behind cases that have not received a diagnosis. The sample in this study was quite similar to ours, as the mean age was 21.73 years, while ours was 20, around 70% of participants were women in both studies, and both studies found that around 70% were in the normal weight range [17]. However, it is noteworthy that we did find significant differences in the prevalence of depression in both sexes, with women being more likely to suffer from this disorder, as is the case in the rest of the world [1]. According to the World Health Organization’s World Mental Health International College Student Project, which was conducted to determine the prevalence of depression among university students in different countries, rates were also found to be around 20% [4]. This study also used a self-administered questionnaire to determine the prevalence, which leads us to believe that there may be a considerable percentage of students without a professional diagnosis.

Our study showed that those students who lived in rented flats or with flatmates, and in general outside the family home, had lower rates of compliance with fish intake recommendations. This finding is consistent with a study conducted in Portugal that examined the eating habits of displaced and non-displaced students. It was observed that students who had to move had a less healthy diet than those who did not. It is noteworthy that displaced students had a lower intake of fish and legumes, while their intake of fast food was higher than recommended [44]. To try to explain why university students tend to eat inadequately, a study carried out at the University of Huelva observed, firstly, that students perceived the concept of healthy cooking as a laborious and complicated task. This was in addition to other important barriers such as lack of budget, lack of planning, class schedules, etc. [45]. This lack of budget may be the reason why we observed in our study that depressed students who are looking for work and studying at the same time, and, collectively, the unemployed, tended to comply less with fish consumption recommendations.

In this study, we have seen that fish consumption follows different patterns across the Spanish geography. In a previous study, it was also observed that the quality of the Spanish diet varies by autonomous community. We have observed coincidences between the universities where there is a higher frequency of fish consumption, and the autonomous communities with better indices of the quality of the diet according to a study carried out by Norte-Navarro et al., such as Cantabria, where they found lower rates of unhealthy eating, while we observed that the universities belonging to these communities are where greater compliance with recommendations was observed. On the other hand, in the Valencian Community, and in Andalusia, Castilla-La Mancha, and Extremadura, higher rates of unhealthy eating were observed, and, in addition, in this study, we found that the universities belonging to these communities were where less compliance with fish intake recommendations was observed [46].

In this study, it has been found that there is a relationship between low fish consumption and the presence of depression, which is consistent with other studies and previously conducted meta-analyses. In 2016, Li et al. conducted a meta-analysis where they also found that higher fish consumption could be related to a lower risk of depression, although significance was only found in those studies conducted in Europe, and the required dose for this association was not conclusive [47]. Another meta-analysis of prospective studies found that there is, indeed, a slight relationship between higher fish consumption and a lower risk of depression, and this association was stronger in women, probably due to the interaction of certain hormones with fish components. However, this study did not find that fish consumption had greater benefits depending on gender [48].

In the same meta-analysis, omega-3 fatty acids were also individually considered, and they were also associated with a lower risk of depression. This could possibly be due to several mechanisms, including its anti-inflammatory effect, its promoting of the fluidity of cell membranes, its facilitating of the transport of certain neurotransmitters, such as serotonin or dopamine, and its improving of the density of receptors, such as dopamine, which has been found to be increased in these patients. Additionally, it has been observed that omega-3s may have a protective effect against oxidative stress, which is altered in patients with depression [20,48]. Even supplementation with omega-3 in patients with depression has had positive results [48]. In another meta-analysis conducted in 2016, it was found that the relationship was significant from a consumption of 50 g of fish per day while, for omega-3s, the relationship was significant, with a consumption of more than 1.8 g per day [30]. On the other hand, fish also contains good quality proteins that help with the metabolism of vitamin D, also present in fish. This vitamin acts jointly with omega-3s, which participate in the synthesis of neurotransmitters and regulation of the circadian rhythm, among other functions [20,48]. However, despite these data, the results of studies that observed fish consumption with depressive symptoms are not conclusive [49].

Other trials have shown that healthy dietary patterns, in addition to the Mediterranean diet, help with both the prevention and treatment of depression. In a study where the intervention consisted of implementing the Mediterranean diet reinforced with fish oil supplements (DHA and EPA), significant improvements were produced in the symptomatology of the disorder [50].

In 2021, a study reported that most of the students surveyed had an inadequate diet or needed changes [51]. In this study, it has been observed that almost 70% of the students had a maximum intake of fish twice a week, while recommendations indicate that adequate consumption is between 2 and 3 servings per week. Therefore, it was observed that most of the sample tended towards low fish intake.

Despite finding a certain relationship between fish consumption and depression, there are many other factors that have more influence on the development of the disorder in a university student, such as age, consumption of certain drugs, sleep habits, self-esteem, or whether they have a partner or not [17]. Therefore, when developing prevention strategies, all of these factors should be taken into account first, and recommendations for fish consumption should be included in a healthy diet for more effective prevention.

### Limitations

The elaboration of this study has had several limitations. Firstly, the response rate was low which makes it more likely to have suffered from sampling or selection bias, which can limit the generalizability of findings to other groups and/or affect the internal validity. In order to combat this, the survey was conducted through a medium available to all students (institutional email) and remained open until the end on the academic year. Other strategies that have been used to increase the participation in online surveys, such as pre-contacting participants or offering compensation, was not possible due to ethical and data protection restraints. In this study, it was not possible to compare respondents and non-respondents by their sociodemographic characteristics given the absence of information for the non-respondents, but it can be said that women were over-represented, which could have introduced some bias in the results.

Furthermore, social desirability or acquiescence bias, which would lead students to report higher intakes of fish and downplay mental health problems, must be considered. However, given the characteristics of the questionnaire and its all-encompassing nature, it is believed that the results of this study show little bias and the results for both fish intake and prevalence of mental health problems are comparable to those reported by other studies.

Regarding fish intake, the type of fish ingested by the students was not taken into account; it would be important for future research to take this into account since different fish have a different composition and can have a different influence on health. Furthermore, the quantity of fish consumed weekly by the students is unknown as only the frequency of consumption was considered. The size of the serving consumed in each instance and by different students could vary significantly. However, fish intake recommendations are most commonly presented as a frequency of consumption and there is no specific amount of fish that is usually recommended and establishing a threshold for adequate/inadequate intake may be difficult. In any case, future studies could collect information on both the frequency and quantity of fish intake in order to provide a more detailed picture of the consumption patterns of the population.

It would be convenient, in future studies, to take into account not only those people who have been diagnosed by a professional, but also to carry out a self-fillable questionnaire to know the prevalence in a more accurate way, since many suffering from depression have not received their diagnosis.

Another of the limitations of this study is the effect of the COVID-19 pandemic on the mental health problems and nutritional behaviors of young people. In this study, no separation has been made between pre/during/post COVID-19 and data from all the academic years has been pooled and treated as a single set of data. Future studies should aim to determine the effect of the COVID-19 pandemic on the prevalence of mental health problems in university students, to determine the effect of the COVID-19 pandemic on the diet of university students and to determine if there is an independent relation among these. This was not attempted in this study, as it fell outside the stated main objective and warrants an independent evaluation.

## 5. Conclusions

The prevalence of depression among the studied sample of Spanish university students is around 10.5%. Students with depression were more likely to be older, working and with a BMI outside the normal limits. The most common frequency of fish consumption was 1 or 2 times per week. Students from northern universities consumed fish more often. Non-compliance with fish intake recommendations significantly elevated the risk of depression. However, since depression is multifactorial, there are other personal factors that may have influenced its onset. Nevertheless, given the results and the modifiable nature of dietary intake patterns, it is important to develop strategies and recommendations to promote fish consumption as a prevention strategy against depression among university students.

## Figures and Tables

**Table 1:** Geographic distribution of participating students.

Location	*n* (%)	95% CI
Total	11,485 (100)	99.95–100
North *	4688 (40.8)	39.91–41.72
South **	6797 (59.2)	58.27–60.08
Valencia	2057 (17.9)	17.21–18.62
Alicante	974 (8.5)	7.98–9.00
Cantabria	103 (0.9)	0.73–1.08
Castilla-La Mancha	300 (2.6)	2.33–2.9
Granada	2973 (25.9)	25.08–26.69
Huelva	503 (4.4)	4.01–4.77
Jaén	290 (2.5)	2.24–2.83
León	1007 (8.8)	8.29–9.30
Salamanca	1462 (12.7)	12.12–13.35
Valladolid	616 (5.4)	4.96–5.79
Vigo	1200 (10.4)	9.89–11.03

* Northern universities: Cantabria, Castilla-La Mancha, León, Vigo, Salamanca and Valladolid. ** Southern universities: Granada, Jaén, Huelva, Alicante and Valencia.

**Table 2:** Sociodemographic characteristics of the students according to depression diagnosis.

	Total (*n* = 11,485)		Depression (*n* = 1207; 10.5%; 9.95–11.08)		No Depression (*n* = 10,278; 89.5%; 88.91–90.04)		*p*-Value *
	*n* (%)/ $\bar{x}$ ± SD	95% CI	*n* (%)/ $\bar{x}$ ± SD	95% CI	*n* (%)/ $\bar{x}$ ± SD	95% CI	
**Sex**							
Male	3138 (27.3)	26.5–28.14	191 (15.8)	13.8–18.03	2947 (28.7)	27.80–29.56	0.001
Female	8347 (72.7)	71.85–73.49	1016 (84.2)	81.96–86.16	7331 (71.3)	70.44–72.19	
**Age**							
Male	20.47 ± 5.17	20.32–20.62	21.28 ± 5.03	20.63–21.93	20.42 ± 5.18	20.26–20.59	0.001
Female	19.97 ± 4.38	19.92–20.02	20.97 ± 5.27	20.84–21.10	19.83 ± 4.23	19.78–19.88	
≤18 years	5937 (51.7)	50.77–52.61	476 (39.4)	36.67–42.26	5461 (53.13)	52.16–54.10	0.001
>18 years	5548 (48.3)	47.38–49.22	731 (60.5)	57.73–63.32	4817 (46.86)	45.89–47.83	0.001
**BMI**							
Underweight	1147 (10.1)	9.57–10.69	156 (13.2)	11.36–15.31	991 (9.8)	9.19–10.35	0.001
Normalweight	8114 (71.6)	71.59–72.42	785 (66.5)	63.74–69.2	7329 (72.2)	71.30–73.05	0.001
Overweight	1651 (14.6)	13.92–15.23	172 (14.6)	12.63–16.75	1479 (14.6)	13.88–15.26	0.983
Obesity	421 (3.7)	3.37–4.08	67 (5.7)	4.45–7.11	354 (3.5)	3.14–3.85	0.001
**Housing**							
Family home	5213 (45.4)	44.55–46.3	528 (43.7)	42.85–44.63	4685 (45.6)	45.27–45.88	0.200
University residence	1303 (11.3)	10.77–11.94	117 (9.7)	9.17–10.23	1186 (11.5)	11.34–11.73	0.060
Rental	4510 (39.3)	38.98–39.33	496 (41.1)	40.21–41.97	4014 (39.1)	38.75–39.35	0.180
Own home/other	459 (3.8)	3.88–4.11	66 (5.5)	5.07–5.88	393 (3.8)	3.7–3.94	0.005
**Coexistence**							
Parents	4845 (42.2)	41.89–42.47	466 (38.6)	37.73–39.48	4379 (42.6)	42.3–42.9	0.007
Roommates	4883 (42.5)	42.23–42.80	479 (39.7)	38.81–40.56	4404 (42.8)	42.54–43.15	0.052
Partner/children	756 (6.58)	6.44–6.72	138 (11.4)	10.87–12.01	618 (6.0)	5.86–6.16	0.001
Alone	1001 (8.7)	8.55–8.88	124 (10.3)	9.74–10.83	877 (8.5)	8.36–8.7	0.038
Accompanied	10,484 (91.28)	90.75–91.79	1083 (89.72)	87.84–91.35	9401(91.46)	90.90–91.99	0.043
**Marital status**							
Married	180 (1.6)	1.49–1.64	50 (4.1)	3.79–4.51	160 (1.6)	1.48–1.63	0.700
Divorced/separated	44 (0.4)	0.28–0.51	10 (0.80)	0.42–1.57	36 (0.35)	0.24–0.49	0.024
Domestic partner	844 (7.3)	7.19–7.50	96 (8.0)	7.48–8.45	748 (7.3)	7.12–7.43	0.350
Single	10,404 (90.6)	90.41–90.75	1079 (89.4)	88.82–89.9	9325 (90.7)	90.54–90.90	0.142
Widowed	11 (0.1)	0.07–0.11	2 (0.16)	0.16–0.26	9 (0.08)	0.07–0.108	0.643
**Employment status**							
Studying and looking for a job	2632 (22.9)	22.67–23.16	378 (31.3)	30.49–32.15	2254 (21.9)	21.67–22.18	0.679
Studying and full-time job	300 (2.6)	2.52–2.70	36 (3.0)	2.68–3.3	246 (2.39)	2.30–2.48	0.210
Studying and part-time job	934 (8.1)	7.97–8.29	115 (9.5)	9.01–10.06	819 (8.0)	7.8–8.13	0.066
Only studying, not looking for a job	7619 (66.3)	66.06–66.61	678 (56.2)	55.28–57.05	6941 (67.5)	67.24–67.81	0.001
**Employment**							
Unemployed	10,251 (89.2)	89.07–89.43	1056 (87.5)	86.88–88.07	9195 (89.4)	89.27–89.64	0.042
Employed	1234 (10.7)	10.56–10.92	151 (12.5)	10.72–14.54	1065 (10.6)	10.17–10.55	0.021

* *p*-Value: ANOVA test.

**Table 3:** Risk of depression according to fish intake recommendation compliance.

	Compliant	Non-Compliant	Attributable Fraction
OR crude	1 Ref.	1.47 (1.31–1.67)	32.0% (23.7–40.1)
ORa Sex	1 Ref.	1.47 (1.30–1.66)	32.0% (23.1–39.8)
ORa Sex, age	1 Ref.	1.44 (1.27–1.63)	30.6% (21.3–38.7)
ORa Sex, age, BMI	1 Ref.	1.44 (1.27–1.63)	30.6% (21.3–38.7)
ORa Sex, age, BMI, housing	1 Ref.	1.43 (1.26–1.62)	30.1% (20.6–38.3)
ORa Sex, age, BMI, housing, coexistence	1 Ref.	1.45 (1.28–1.65)	31.0% (21.9–39.4)
ORa Sex, age, BMI, housing, coexistence, marital status	1 Ref.	1.44 (1.27–1.63)	30.6% (21.3–38.7)
ORa Sex, age, BMI, housing, coexistence, marital status, employment	1 Ref.	1.45 (1.28–1.64)	31.0% (21.9–39.0)

## Data Availability

The data presented in this study are available upon reasonable request from the corresponding author. The data are not publicly available due to personal data protection.

## Supplementary Materials

The following supporting information can be downloaded at: https://www.mdpi.com/article/10.3390/nu15122757/s1, Table S1. Compliance with recommendations on the frequency of consumption of fish by universities and according to geographical distribution. Table S2. Compliance with fish consumption recommendations in depressed and non-depressed individuals based on population characteristics.
