# Supplementary material for: Prevalence of Depression and Fish Consumption among First Year Spanish University Students: UniHcos Project
Source: Nutrients. 2023 Jun 15;15(12):2757. doi: 10.3390/nu15122757 (PMC10300875; doi:10.3390/nu15122757)
Supplement: Supplementary file 1 [file nutrients-15-02757-s001.zip › nutrients-2425299-supplementary.pdf]

Supplementary Table S1. Compliance with recommendations on the frequency of consumption of fish by universities and according to geographical distribution.

		Yes		1-2 times/week		3-4 times/week		Daily		No		<1 times/week		Never/rarely						
	n (%)	n (%)	95% CI	n (%)	95% CI	n (%)	95% CI	n (%)	95% CI	n (%)	95% CI	n (%)	95% CI	n (%)	95% CI	Per capita consumption (kg)	Ratio*	p-value[1]	p-value[2]	p-value[3]
**Total**	11485 (100)	7723 (67.2)	66.37-68.10	5081 (44.2)	43.30-45.15	2383 (20.7)	20.01-21.50	259 (2.3)	1.99-2.54	3762 (32.7)	31.89-33.62	2267 (19.7)	19.01-20.48	1495 (13.0)	12.40-13.64					
**Universities**																				
**Valencia**	2057 (100)	1348 (65.5)	63.42-67.57	922 (44.8)	42.66-47.00	386 (18.7)	17.11-20.53	40 (1.9)	1.41-2.66	709 (34.5)	34.46-36.57	395 (19.2)	17.63-20.98	314 (15.3)	13.75-16.90	20.83	3.14	0.001	0.001	0.001
**Alicante**	974 (100)	686 (70.4)	67.43-73.26	426 (43.7)	40.60-46.92	225 (23.1)	20.51-25.90	35 (3.6)	2.55-5.01	288 (29.5)	26.73-32.56	175 (17.9)	15.63-20.55	113 (11.6)	15.63-20.55		3.37	0.001	0.001	1
**Cantabria**	103 (100)	77 (74.7)	65.06-82.56	46 (44.7)	34.96-54.75	26 (25.2)	17.43-34.93	5 (4.8)	1.8-11.5	26 (25.2)	17.43-34.93	13 (12.6)	7.15-20.96	13 (12.6)	7.16-20.96	24.09	3.10	0.001	0.001	1
**Castilla-La Mancha**	300 (100)	197 (65.5)	59.95-70.96	110 (36.6)	31.25-42.42	74 (24.6)	19.97-30.02	13 (4.3)	2.42-7.47	103 (34.3)	29.03-40.04	58 (19.3)	15.11-24.35	45 (15.0)	11.25-19.66	22.29	2.9	0.001	0.001	0.159
**Granada**	2973 (100)	1971 (66.3)	64.56-67.99	1340 (45.1)	43.27-46.88	573 (19.3)	17.87-20.74	58 (1.9)	1.49-2.5	1002 (33.7)	32.00-35.43	639 (21.5)	20.03-23.02	363 (12.2)	11.06-13.45		3.11	0.001	0.001	0.001
**Huelva**	503 (100)	307 (61.0)	56.60-65.29	208 (41.3)	37.03-45.80	90 (17.9)	14.69-21.58	9 (1.8)	0.87-3.49	196 (38.9)	34.70-43.39	123 (24.4)	24.45-28.49	73 (14.5)	11.61-17.96	21.28	2.86	0.001	0.001	0.001
**Jaen**	290 (100)	206 (71.0)	65.38-76.11	143 (49.3)	43.43-55.20	55 (18.9)	14.71-24.05	8 (2.7)	1.28-5.57	84 (28.9)	23.88-34.61	59 (20.3)	15.95-25.53	25 (8.6)	5.76-12.61		3.33	0.001	0.001	0.001
**Leon**	1007 (100)	696 (69.1)	66.14-71.94	450 (44.7)	41.59-47.82	222 (22)	19.54-24.76	24 (2.4)	1.56-3.58	311 (30.9)	28.05-33.85	200 (19.9)	17.46-22.48	111 (11.0)	9.18-13.16		2.56	0.001	0.001	0.001
**Salamanca**	1462 (100)	901 (61.6)	59.07-64.12	576 (39.4)	36.89-41.96	297 (20.3)	18.29-22.48	28 (1.9)	1.3-2.79	561 (38.4)	35.87-40.92	304 (20.8)	18.75-22.98	257 (17.6)	15.68-19.64	26.95	2.28	0.001	0.001	0.027
**Valladolid**	616 (100)	473 (76.8)	73.20-80.02	282 (45.8)	41.80-49.80	178 (28.9)	25.37-32.68	13 (2.1)	1.17-3.6	143 (23.2)	19.90-26.80	86 (13.9)	11.3-17.00	57 (9.3)	7.13-11.89		2.84	0.001	0.001	0.009
**Vigo**	1200 (100)	861 (71.7)	69.09-74.26	578 (48.2)	45.30-51.03	257 (21.4)	19.14-23.87	26 (2.1)	1.44-3.2	339 (28.2)	25.73-30.90	215 (17.9)	15.81-20.23	124 (10.3)	8.69-12.22	26.54	2.64	0.001	0.001	0.001
**Universities distribution**																				
**North***	4688 (100)	3205 (68.4)	67.00-69.69	2042 (43.6)	42.13-44.99	1054 (22.5)	21.30-23.71	109 (2.3)	1.92-2.8	1483 (31.6)	30.30-32.99	876 (18.7)	17.58-19.83	607 (12.9)	12.00-13.95	24.95	2.74	0.001	0.001	0.001
**South****	6797 (59.2)	4518 (66.4)	65.33-67.59	3039 (44.7)	43.52-45.90	1329 (19.5)	18.62-20.52	150 (2.2)	1.87-2.59	2279 (33.5)	32.40-34.66	1391 (20.4)	19.51-21.44	888 (13.1)	12.27-13.89	21.05	3.15	0.001	0.001	0.001

*Northern universities: Cantabria, Castilla- La mancha, León, Vigo, Salamanca y Valladolid.
**Southern universities: Granada, Jaén, Huelva, Alicante y Valencia.
Ratio: percentage who meet the recommendations (YES)/Per capita consumption
p-value: ANOVA test.

p-value [1]: p-value between those who meet the recommendations (YES) and those who do not (NO).
p-value [2]: p-value between those who meet the recommendations (daily, 1-2times/week, 3-4 times/week).
p-value [3]: p-value between those who do not meet the recommendations (<1time/week, never/rarely).

Supplementary Table S2. Compliance with fish consumption recommendations in depressed and non-depressed individuals based on population characteristics.

	Depression						No depression					
	Total (n=1207)	Compliant		Non-compliant		p-value*	Total (n=10278)	Compliant		Non-compliant		p-value*
	N (%)	N (%)	95% CI	N (%)	95% CI		N (%)	N (%)	95% CI	N (%)	95% CI	
**Total**	1207 (100)	715 (59.23)	56.39-62.01	492 (40.76)	37.98-43.60	0.001	10278 (100)	7008 (68.18)	67.27-69.08	3270 (31.81)	30.91-32.72	0.001
**Age**												
<18 years	476 (100)	295 (61.97)	57.97-66.32	181 (38.02)	33.67-42.57	0.001	5461 (100)	3818 (69.9)	68.67-71.12	1643 (30.1)	28.87-31.32	0.001
>18 years	731 (100)	420 (57.45)	53.77-61.05	311 (42.54)	38.94-46.22	0.001	4817 (100)	3190 (66.22)	64.86-67.55	1627 (33.77)	32.44-35.13	0.001
**Sex**												
Male	191 (100)	103 (53.92)	46.59-61.10	88 (46.07)	38.89-53.40	0.124	2947 (100)	2016 (68.40)	66.68-70.07	931 (31.59)	29.92-33.31	0.001
Female	1016 (100)	612 (60.23)	57.14-63.25	404 (39.76)	36.75-42.85	0.001	7331 (100)	4992 (68.09)	67.01-69.15	2339 (31.9)	30.84-32.98	0.001
**BMI**												
Underweight	156 (100)	96 (61.53)	53.38-69.10	60 (38.46)	30.89-46.61	0.001	991 (100)	667 (67.30)	64.27-70.20	324 (32.69)	29.79-35.72	0.001
Normal weight	785 (100)	469 (59.74)	56.21-63.18	316 (40.25)	36.81-43.78	0.001	7329 (100)	5029 (68.61)	67.53-69.67	2300 (31.38)	30.32-32.46	0.001
Overweight	172 (100)	97 (56.39)	48.63-63.86	75 (43.60)	36.14-51.36	0.017	1479 (100)	1006 (68.01)	65.56-70.37	473 (31.98)	29.62-34.43	0.001
Obesity	67 (100)	37 (55.22)	42.63-67.21	30 (44.77)	32.78-57.36	0.226	354 (100)	230 (64.97)	59.72-69.89	124 (35.03)	30.10-40.27	0.001
**Housing**												
Family home	528 (100)	349 (66.09)	61.86-70.09	179 (33.90)	29.90-38.13	0.001	4685 (100)	3469 (74.04)	72.75-75.29	1216 (25.95)	24.70-27.24	0.001
University residence	117 (100)	74 (63.24)	53.78-71.82	43 (36.75)	28.17-46.21	0.001	1186 (100)	886 (74.70)	72.11-77.13	300 (25.29)	22.86-27.88	0.001
Rental	496 (100)	248 (50)	45.51-54.48	248 (50)	45.51-54.48	1	4014 (100)	2368 (58.99)	57.45-60.51	1646 (41.00)	39.48-42.54	0.001
Own home/other	66 (100)	44 (66.66)	53.88-77.49	22 (33.33)	22.50-46.12	0.001	393 (100)	285 (72.51)	67.77-76.81	108 (27.48)	23.18-32.23	0.001
**Coexistence**												

Parents	466 (100)	310 (66.52)	62.01-70.76	156 (33.47)	29.23-37.99	0.001	4379 (100)	3270 (74.67)	73.35-75.95	1109 (25.25)	23.98-26.57	0.001
Roommates	479 (100)	254 (53.03)	48.44-57.55	225 (46.97)	42.44-51.55	0.060	4408 (100)	2726 (61.84)	60.38-63.27	1678 (38.07)	36.63-39.52	0.001
Partner/children	138 (100)	82 (59.42)	50.71-67.59	56 (40.57)	32.40-49.28	0.001	618 (100)	423 (68.44)	64.59-72.06	195 (31.55)	27.93-35.40	0.001
Alone	124 (100)	69 (55.64)	46.46-64.47	55 (44.35)	35.52-53.53	0.075	877 (100)	589 (67.16)	63.92-70.24	288 (32.83)	29.75-36.07	0.001
Accompanied	1083 (100)	646 (59.64)	56.65-62.57	437 (40.35)	37.42-43.34	0.001	9401 (100)	6419 (68.28)	67.32-69.21	2982 (31.72)	30.78-32.67	0.001
**Marital status**												
Married	20 (100)	13 (65.00)	40.94-83.69	7 (35.00)	16.30-59.05	0.057	160 (100)	129 (80.62)	73.47-86.27	31 (19.37)	13.72-26.53	0.001
Divorced/Separated	10 (100)	4 (40)	13.69-72.63	6 (60.00)	27.36-86.30	0.371	27 (100)	21 (77.77)	57.26-90.62	6 (22.22)	9.37-42.73	0.001
Domestic partner	95 (100)	61 (64.21)	53.65-73.60	34 (35.78)	26.39-46.34	0.001	748 (100)	506 (67.64)	64.14-70.96	242 (32.35)	29.03-35.85	0.001
Single	1079 (100)	634 (58.75)	55.74-61.70	445 (41.24)	38.29-44.25	0.001	9325 (100)	6338 (67.96)	67.00-68.91	2987 (32.03)	31.08-32.99	0.001
Widowed	6 (100)	6 (100)	51.68-100.00	0 (0)	0.00-48.31	0.003	5 (100)	1 (20.00)	1.05-70.12	4 (80.00)	29.87-98.94	0.205
**Employment status**												
Studying and looking for a job	378 (100)	206 (54.49)	49.32-59.57	172 (45.50)	40.42-50.67	0.013	2254 (100)	1489 (66.06)	64.05-68.00	765 (33.93)	31.99-36.94	0.001
Studying and full-time job	36 (100)	22 (61.11)	43.52-76.37	14 (38.88)	23.62-56.47	0.059	264 (100)	202 (76.51)	70.84-81.39	62 (23.48)	18.60-29.15	0.001
Studying and part-time job	115 (100)	77 (66.95)	57.48-75.27	38 (33.04)	24.72-42.51	0.001	819 (100)	545 (66.54)	63.18-69.75	274 (33.45)	30.24-36.81	0.001
Only studying, not looking for a job	668 (100)	410 (61.37)	57.55-65.06	258 (38.62)	34.93-42.44	0.001	6941 (100)	4772 (68.75)	67.64-69.83	2169 (31.24)	30.16-32.35	0.001
**Employment**												
Unemployed	1056 (100)	616 (58.33)	55.28-61.31	440 (41.66)	38.68-44.71	0.001	9195 (100)	6261 (68.09)	67.12-69.04	2934 (31.9)	30.95-32.87	0.001
Employed	151 (100)	99 (65.56)	57.34-72.97	52 (34.43)	27.02-42.65	0.001	1083 (100)	747 (68.97)	66.10-71.70	336 (31.02)	28.29-33.89	0.001

*Meets the recommendations: students who eat fish between 1-2 times per week or more.

*Does not meet the recommendations: students who eat fish less than 1 times per week or less.

p-value: ANOVA test.

OR: between students with and without depression and the adequacy of fish consumption recommendations.
